# Prevalence of Atrial Fibrillation and Antithrombotic Therapy in Hemodialysis Patients: Cross-Sectional Results of the Vienna InVestigation of AtriaL Fibrillation and Thromboembolism in Patients on HemoDIalysis (VIVALDI)

**DOI:** 10.1371/journal.pone.0169400

**Published:** 2017-01-04

**Authors:** Oliver Königsbrügge, Florian Posch, Marlies Antlanger, Josef Kovarik, Renate Klauser-Braun, Josef Kletzmayr, Sabine Schmaldienst, Martin Auinger, Günther Zuntner, Matthias Lorenz, Ella Grilz, Gerald Stampfel, Stefan Steiner, Ingrid Pabinger, Marcus Säemann, Cihan Ay

**Affiliations:** 1 Clinical Division of Hematology and Hemostaseology, Department of Medicine I, Medical University of Vienna, Vienna, Austria; 2 Department of Medicine, Clinical Division of Oncology, Medical University of Graz, Austria; 3 Clinical Division of Nephrology, Department of Medicine III, Medical University of Vienna, Vienna, Austria; 4 Department of Medicine VI, Wilhelminenspital, Vienna, Austria; 5 Department of Medicine III, Donauspital, Vienna, Austria; 6 Department of Medicine I, Kaiser-Franz-Josef-Spital, Vienna, Austria; 7 Department of Medicine III, Hietzing Hospital, Vienna, Austria; 8 Department of Medicine I, Rudolfstiftung Hospital, Vienna, Austria; 9 Vienna Dialysis Center, Vienna, Austria; 10 Department of Medicine, Thrombosis and Hemostasis Program, McAllister Heart Institute, University of North Carolina at Chapel Hill, Chapel Hill, NC, United States of America; Chang Jung Christian University, TAIWAN

## Abstract

**Background:**

Atrial fibrillation (AF) adds significant risk of stroke and thromboembolism in patients on hemodialysis (HD). The aim of this study was to investigate the prevalence of AF in a population-based cohort of HD patients and practice patterns of antithrombotic therapy for stroke prevention in AF.

**Methods:**

The Vienna InVestigation of AtriaL fibrillation and thromboembolism in patients on hemodialysis (VIVALDI), an ongoing prospective observational cohort study, investigates the prevalence of AF and the risk of thromboembolic events in HD patients in Vienna, Austria. We analyzed cross-sectional data of 626 patients (63.4% men, median age 66 years, approx. 73% of HD patients in Vienna), who provided informed consent. A structured interview with each patient was performed, recent and archived ECGs were viewed and medical histories were verified with electronic records.

**Results:**

The overall prevalence of AF was 26.5% (166 patients, 71.1% men, median age 72 years) of which 57.8% had paroxysmal AF, 3.0% persistent AF, 32.5% permanent AF, and 6.6% of patients had newly diagnosed AF. The median CHA_2_DS_2_-VASc Score was 4 [25^th^-75^th^ percentile 3–5]. In multivariable analysis, AF was independently associated with age (odds ratio: 1.05 per year increase, 95% confidence interval: 1.03–1.07), male sex (1.7, 1.1–2.6), history of venous thromboembolism (2.0, 1.1–3.6), congestive heart failure (1.7, 1.1–2.5), history of or active cancer (1.5, 1.0–2.4) and time on HD (1.08 per year on HD, 1.03–1.13). Antithrombotic treatment was applied in 84.4% of AF patients (anticoagulant agents in 29.5%, antiplatelet agents in 33.7%, and both in 21.1%). In AF patients, vitamin-K-antagonists were used more often than low-molecular-weight heparins (30.1% and 19.9%).

**Conclusions:**

The prevalence of AF is high amongst HD patients and is associated with age, sex, and distinct comorbidities. Practice patterns of antithrombotic treatment indicate a lack of consensus for stroke prevention in HD patients with AF.

## Introduction

Atrial fibrillation (AF) is a common cardiac arrhythmia that affects 1–2% of the general population, and increases the risk of stroke [1,2]. In patients with end-stage renal disease (ESRD) receiving hemodialysis (HD), AF is an underestimated comorbidity. Although it is recognized as a causal factor for thromboembolic ischemic stroke and is associated with increased mortality in HD patients [3,4], stroke prevention using antithrombotic agents is complicated by an excessive risk of bleeding in ESRD patients [5]. In the general population, stroke prevention in AF with oral anticoagulation with vitamin-K-antagonists (VKA) can reduce the incidence of stroke by 60% [6]. However, in HD patients there is currently no clear evidence for how to achieve stroke prevention [7], and large differences exists in the current practice for use of antithrombotic agents in HD patients [8].

The reported prevalence of AF in HD patients is 3.8 to 27% [9–14]. This large variability in AF prevalence may be explained by regional differences [8], but may also be biased by the different study designs and data capture methods. The overall higher prevalence of AF compared to the general population is hypothesized to be associated with the HD procedure itself,[15] which can lead to an increase of cardiac dimensions and decrease of ejection fraction, resulting in AF development [12]. Thus, the prevalence of AF in HD patients may depend upon hemodialysis-specific patient characteristics as well as on treatment modalities.

In this population-based cohort of HD patients, we investigated the prevalence of AF, analyzed the association of AF with clinico-epidemiological factors, and collected data on practice patterns of antithrombotic treatment strategies applied in HD patients for stroke prevention in AF.

## Patients and Methods

The Vienna InVestigation of AtriaL fibrillation and thromboembolism in hemDIialysis patients (VIVALDI) is a cohort study with aims to gather population-based epidemiologic data on the prevalence of AF, thromboembolic events and use of antithrombotic treatments in HD patients and prospective data on the incidence of stroke and thromboembolism, as well as the incidence of new AF, bleeding, hospitalization, cardiovascular events, thrombotic shunt complications, and mortality. The study consists of a cross-sectional baseline investigation and a prospective observational evaluation of study outcomes and has approval of the local ethics committees. VIVALDI is conducted in accordance with the declaration of Helsinki and its later amendments.

**Fig 1:**
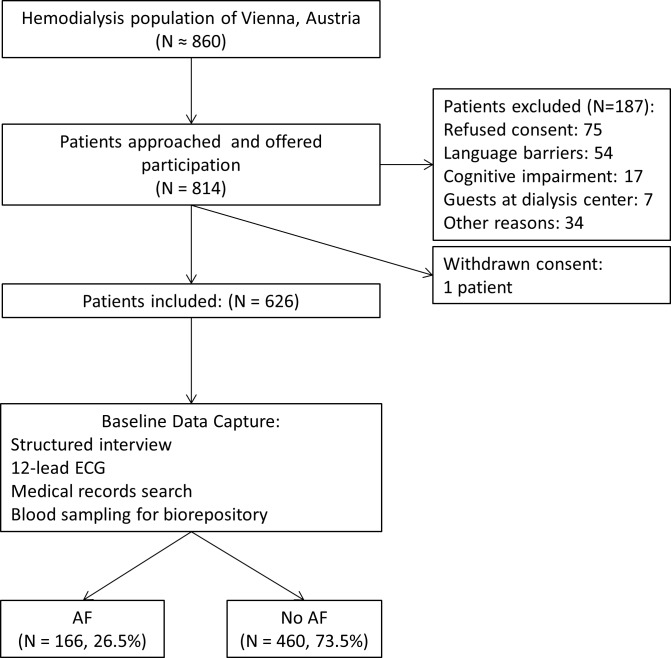
Flow chart of the patient recruitment.

Patient treated at the seven major HD centers in the city of Vienna, Austria, were eligible for recruitment. The study cohort was recruited between April 2014 and July 2015. Patients aged 18 years or above, with an independent diagnosis of ESRD requiring long-term ambulatory HD treatment, able to provide signed and dated informed consent, and willing to comply with all study procedures were eligible for inclusion. Patients were excluded, if they were pregnant, lactating, suspected of pregnancy, incapable to consent, hospitalized as an in-patient, or serving a prison term at the time of enrolment. The steps of patient enrolment and corresponding patient numbers are provided in **Fig 1.** Out of approximately 860 patients receiving HD in Vienna (population of 1.7 million in 2014–2015), Austria, 814 (94.6%) were personally approached and 626 patients (~73% of the total HD population in the city) met the inclusion criteria and consented to participate. The prospective observation period is still ongoing.

A trained study investigator interviewed each patient individually and recorded demographics, medical histories and HD parameters using the REDCap electronic data capture tools hosted at the Medical University of Vienna, Department of Medicine I, Clinical Division of Hematology and Haemostaseology [16]. Data quality was verified with treating nephrologists and the medical documentation of the participating dialysis centers. Cause of ESRD was established from the medical records at the dialysis center and classified according to categories of the Austrian Dialysis and Transplantation Registry [17]. A diagnosis of AF was recorded for symptomatic or asymptomatic AF in any one of the following (1) signs of AF on a recent 12-lead resting ECG within one month of recruitment, (2) documented AF episode during previous ECGs conducted routinely or in case of arrhythmia at the dialysis centers, or (3) recorded diagnosis of AF in medical records. Recently diagnosed AF was defined as recent new diagnosis of AF, where a specific type had not been established. Paroxysmal AF is recurrent self-terminating AF. Persistent AF is long-lasting AF that requires termination by cardioversion. In permanent AF, patients do not return to sinus rhythm or attempt cardioversion [18]. Time on HD was calculated as the sum of the current HD treatment period and periods of HD treatment before renal transplant in patients where applicable. Inter-dialytic weight gain was calculated as the difference between pre-dialysis weight and target dry body weight. A history of venous thromboembolism only includes non-catheter-associated deep vein thrombosis and pulmonary embolism.

### Statistical methods

Descriptive parameters of the study population are given as absolute and relative frequencies or median values with 25^th^ to 75^th^ percentile, where appropriate. In order to compare the distribution of patient parameters between patients with and without AF, the dependent variable, we used the Mann-Whitney U test for ordinal and continuous independent variables or chi-squared test for categorical independent variables, where applicable. The odds ratios (OR) for association between AF and patient characteristics and comorbidities were calculated in univariable and multivariable logistic regression models. A two-tailed p-value below 0.05 was regarded as statistically significant. All calculations were conducted with SPSS (IBM SPSS for Windows, Version 23.0. Armonk, NY) and graphs were drawn with GraphPad Prism (GraphPad Software, Version 5.00 for Windows, San Diego CA).

## Results

### Study population

A total of 626 patients were recruited into the study, of which 397 (63.4%) were men. The median age was 66 years (25^th^ to 75^th^ percentile 55–75) and the median BMI was 25.7 kg/m^2^ (22.4–29.6). Diabetic nephropathy was the most frequent cause for ESRD (25.6%) followed by vascular nephropathy (19.3%). Further baseline characteristics of the study cohort are provided in **Table 1**.

**Table 1:** Characteristics of the study population at baseline.

	Full cohort	Non-AF cohort	AF cohort	p-value*
Patients, n (%)	626 (100)	460 (73.5)	166 (26.5)	—
Male sex (%)	397 (63.4)	279 (60.7)	118 (71.1)	0.017
Age, median (25^th^– 75^th^ percentile)	66 (55–75)	63.5 (50–73)	71.5 (64–78)	<0.001
BMI, median (25^th^– 75^th^ percentile)	25.7 (22.4–29.6)	25.6 (22.2–29.4)	25.9 (22.7–29.7)	0.734
Caucasian ethnicity (%)	601 (96)	435 (94.6)	166 (100)	n.a.
African ethnicity (%)	12 (1.9)	12 (2.6)	0	n.a.
Asian, pacific islander ethnicity (%)	13 (2.1)	13 (2.8)	0	n.a.
**Etiology of ESRD, n (%)**				
Diabetic NP	160 (25.6)	117 (25.4)	43 (25.9)	0.906
Vascular NP	121 (19.3)	82 (17.8)	39 (23.5)	0.113
Glomerular nephritis	82 (13.1)	65 (14.1)	17 (10.2)	0.203
Atrophic NP	57 (9.1)	41 (8.9)	16 (9.6)	0.781
Cystic non-hereditary NP	36 (5.8)	26 (5.7)	10 (6.0)	0.860
Hereditary NP	31 (5.0)	26 (5.7)	5 (3.0)	0.179
Nephrectomy	20 (3.2)	10 (2.2)	10 (6.0)	0.016
Iatrogenic/toxic NP	28 (4.5)	19 (4.1)	9 (5.4)	0.491
Other causes	91 (14.5)	74 (16.1)	17 (10.2)	0.067
**Dialysis history, n (%)**				
History of renal transplantation	90 (14.4)	64 (13.9)	26 (15.7)	0.582
Previous peritoneal dialysis	46 (7.3)	35 (7.6)	11 (6.6)	0.606
Current vascular access				
AV fistula	329 (52.6)	244 (53.0)	85 (51.2)	0.684
AV graft	72 (11.5)	57 (12.4)	15 (9.0)	0.246
Central venous catheter	221 (35.3)	157 (34.1)	64 (38.6)	0.307
others	4 (0.7)	2 (0.4)	2 (1.2)	0.286
**Dialysis parameters, median (25th– 75th percentile)**				
Remaining diuresis, ml	500 (0–1000)	500 (0–1000)	350 (0–1000)	0.295
Inter-dialytic weight gain, kg	2.0 (1.1–3.0)	2.1 (1.1–3.1)	1.9 (1.2–2.6)	0.135
Time on hemodialysis, years	2.7 (1.0–5.0)	2.5 (1.0–5.0)	3.0 (1.1–6.0)	0.084
**Comorbidities, n (%)**				
History of stroke or TIA	127 (20.3)	82 (17.8)	45 (27.1)	0.011
History of myocardial infarction	105 (16.8)	69 (15.0)	36 (21.7)	0.048
Coronary heart disease	233 (37.2)	150 (32.6)	83 (50.0)	<0.001
Artificial heart valve	43 (6.9)	22 (4.8)	21 (12.7)	0.001
Bioprosthetic valve	31 (5.0)	16 (3.5)	15 (9.0)	0.004
Mechanical valve	10 (1.6)	5 (1.1)	5 (3.0)	0.071
History of VTE	61 (9.7)	36 (7.8)	25 (15.1)	0.007
Deep vein thrombosis	44 (7.0)	27 (5.9)	17 (10.2)	0.075
Pulmonary embolism	32 (5.1)	18 (3.9)	14 (8.4)	0.037
Peripheral artery disease	197 (31.5)	139 (30.2)	58 (34.9)	0.261
Diabetes	237 (37.9)	168 (36.5)	69 (41.6)	0.244
Hypertension	576 (92.0)	423 (92.0)	153 (92.2)	0.931
Congestive heart failure	184 (29.4)	114 (24.8)	70 (42.2)	<0.001
Cancer history or active	152 (24.3)	96 (20.9)	56 (33.7)	<0.001
Smokers	306 (48.9)	230 (50.0)	76 (45.8)	0.351
**Long-term antithrombotic therapy, n (%)**				
LMWH s.c. on non-HD days	59 (9.4)	26 (5.7)	33 (19.9)	<0.001
20 mg o.d.	4 (0.6)	2 (0.4)	2 (1.2)	<0.001
40 mg o.d.	39 (6.2)	18 (3.9)	21 (12.7)	<0.001
60 mg o.d.	11 (1.8)	5 (1.1)	6 (3.6)	<0.001
80 mg o.d.	5 (0.8)	1 (0.2)	4 (2.4)	<0.001
Vitamin K antagonist	77 (12.3)	27 (5.9)	50 (30.1)	<0.001
Antiplatelet agent	345 (55.1)	254 (55.2)	91 (54.8)	0.930

**Table legend:** AF–atrial fibrillation, BMI–body-mass-index, NP–nephropathy, AV–arteriovenous, TIA–transient ischemic attack, VTE–venous thromboembolism, LMWH–low-molecular-weight heparin, o.d.–once daily * Mann-Whitney U test or chi^2^ p-value for non-AF cohort versus AF cohort.

### AF prevalence

A diagnosis of AF was recorded in 166 patients (26.5%). AF patients were predominantly men (71.1%) with a median age of 71.5 years (64–78). In 59.6% of patients with AF, AF developed after commencing HD treatment, while 40.4% had AF before ESRD. AF was present in 93 patients (56%) as paroxysmal AF, in 5 patients (3%) as persistent AF, in 53 patients (32%) as permanent AF, and in 15 patients (9%) as recently diagnosed or unknown type of AF.

**Fig 2:**
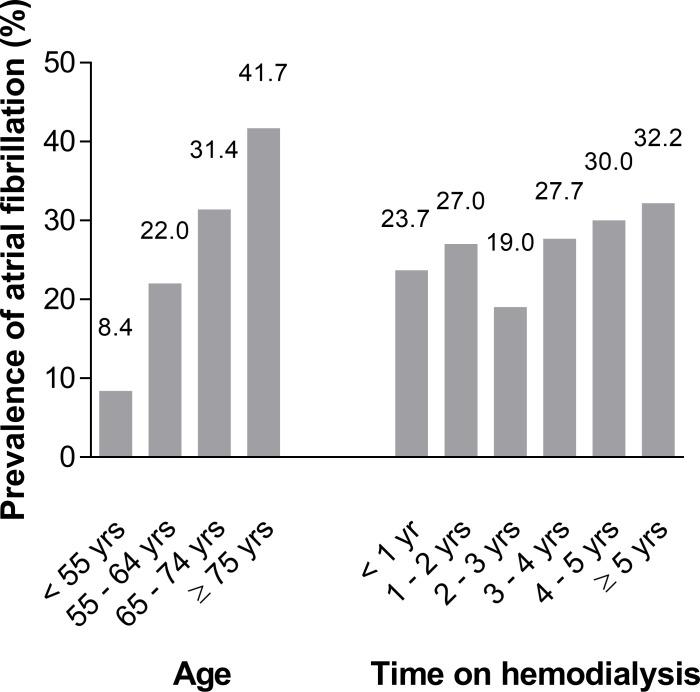
Prevalence of atrial fibrillation in patients with increasing age and time on hemodialysis.

The prevalence of AF rose with increasing age, from 8.4% (14/166) in patients aged younger than 55 years, 22.0% (29/132) in those aged 55–64 years, 31.4% (55/175) in those aged 65–74 years, and 41.7% (68/163) in patients aged ≥ 75 years (**Fig 2**).

The prevalence of AF was 23.7% (40/169) in patients with ≤ 1 year of time on HD, 27.0% (31/115) with 1 to 2 years of time on HD, 19.0% (16/84) with 2 to 3 years time on HD, 27.7% (18/65) with 3 to 4 years time on HD, 30.0% (15/50) with 4 to 5 years of time on HD and 32.2% (46/143) with more than 5 years time on HD (**Fig 2).**

The distribution of AF for the baseline parameters sex, age, time on HD, a history of stroke or transient ischemic attack (TIA), coronary heart disease, venous thromboembolism (VTE), artificial heart valves, active or history of cancer, and congestive heart failure had significant trends in the Wilcoxon-Mann-Whitney U test and chi-squared test (**Table 1**). In univariable logistic regression, these factors were associated with the presence of AF (**Table 2**).

**Table 2:** Factors associated with prevalence of atrial fibrillation Model 1 (N = 626).

Characteristics	Univariable OR (95%CI)	p	Multivariable OR (95% CI)	p
Male sex	1.60 (1.09–2.34)	0.017	1.73 (1.13–2.63)	0.011
Age, per year	1.05 (1.04–1.07)	<0.001	1.05 (1.03–1.07)	<0.001
Time on HD, per year	1.02 (0.98–1.06)	0.370	1.03 (0.99–1.08)	0.135
Stroke/TIA	1.71 (1.13–2.60)	0.011	1.36 (0.86–2.13)	0.188
Coronary heart disease	2.07 (1.44–2.97)	<0.001	1.41 (0.95–2.10)	0.091
Artificial heart valve	2.88 (1.54–5.40)	0.001	1.80 (0.91–3.56)	0.094
VTE	2.09 (1.21–3.60)	0.008	1.99 (1.11–3.59)	0.022
Congestive heart failure	2.21 (1.52–3.22)	<0.001	1.66 (1.10–2.51)	0.015
Cancer history/active	1.93 (1.30–2.86)	0.001	1.55 (1.02–2.36)	0.042

In multivariable logistic regression with covariates identified as potentially associated with AF prevalence, male sex, increased age, history of VTE, cancer, and congestive heart failure were identified as independently associated with increased risk of AF diagnosis (**Table 2**). After excluding patients with AF diagnosed chronologically before ESRD for a second model of 559 patients, increased time on HD was associated with the presence of AF (**Table 3**).

**Table 3:** Model 2 of factors associated with atrial fibrillation, excluding patients with a diagnosis of atrial fibrillation before occurrence of ESRD (N = 559).

Characteristics	Univariable OR (95%CI)	p	Multivariable OR (95%CI)	p
Male sex	1.92 (1.18–3.14)	0.009	2.18 (1.28–3.70)	0.004
Age, per year	1.05 (1.03–1.06)	<0.001	1.05 (1.03–1.07)	<0.001
Time on HD, per year	1.06 (1.02–1.11)	0.003	1.08 (1.03–1.13)	0.001
Stroke/TIA	1.56 (0.93–2.60)	0.090	1.14 (0.66–1.98)	0.631
Coronary heart disease	2.03 (1.31–3.14)	0.002	1.41 (0.87–2.27)	0.160
Artificial heart valve	2.24 (1.02–4.89)	0.043	1.44 (0.62–3.34)	0.400
VTE	1.94 (1.00–3.75)	0.049	1.89 (0.93–3.85)	0.081
Congestive heart failure	1.59 (0.99–2.53)	0.052	1.24 (0.75–2.03)	0.403
Cancer history/active	1.65 (1.02–2.68)	0.043	1.21 (0.72–2.03)	0.481

Risk evaluation for stroke and bleeding in AF patients resulted in a median CHA_2_DS_2_-Vasc Score of 4 (25^th^ to 75^th^ percentile 3–5) and a median HAS-BLED Score of 4 (3–4). A strong indication for anticoagulation was given in 159 patients (95.8% of AF patients) who had a CHA_2_DS_2_-Vasc ≥2.

### Anti-thrombotic therapy

The frequencies of antithrombotic medications in mono- and combination therapy are given in Table 4.

**Table 4:** Practice patterns of antithrombotics in patients on HD.

Antithrombotic therapy	Full cohort, count (% of N = 626)	AF cohort, count (% of N = 166)
Vitamin-K-antagonist	77 (12.3)	50 (30.1)
Monotherapy	51 (8.1)	35 (21.1)
Combination with antiplatelet agent	26 (4.2)	15 (9.0)
Triple therapy (VKA + clopidogrel + aspirin)	3 (0.5)	0
Low-molecular-weight heparin	59 (9.4)	33 (19.9)
Monotherapy	27 (4.3)	14 (8.4)
Combination with antiplatelet agent	32 (5.1)	19 (11.4)
Triple therapy (LMWH + clopidogrel + aspirin)	6 (1.0)	4 (2.4)
Antiplatelet	345 (55.1)	91 (54.8)
Monotherapy	286 (45.7)	56 (33.7)
Dual antiplatelet therapy	50 (8.0)	11 (6.7)
No antithrombotic	203 (32.4)	26 (15.6)

For the dual pathway therapy in the full cohort, LMWH use was preferred over VKA (5.1% versus 4.2%, p = 0.013). The new generation P2Y12 inhibitors, ticagrelor and prasugrel, were not used in dual pathway antithrombotic therapy and direct oral anticoagulants were not used in any patient.

**Fig 3:**
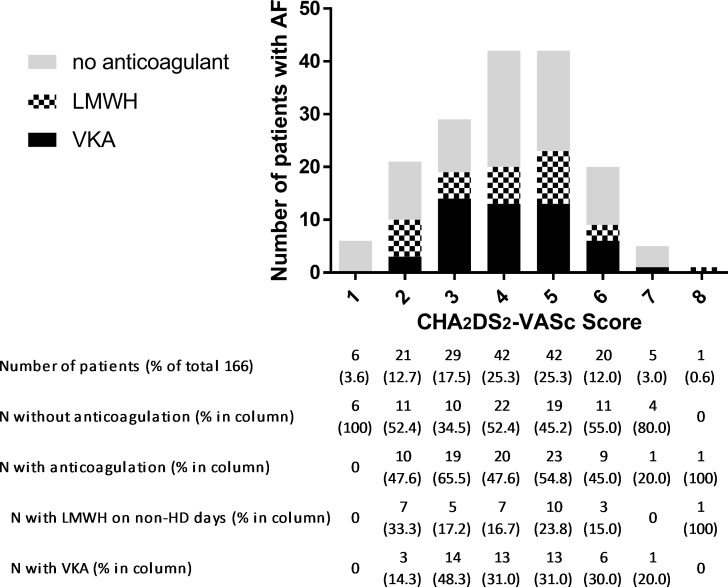
Distribution of patients with AF across CHA2DS2-VASc Scores and frequency of corresponding anticoagulation treatment.

**Fig 4:**
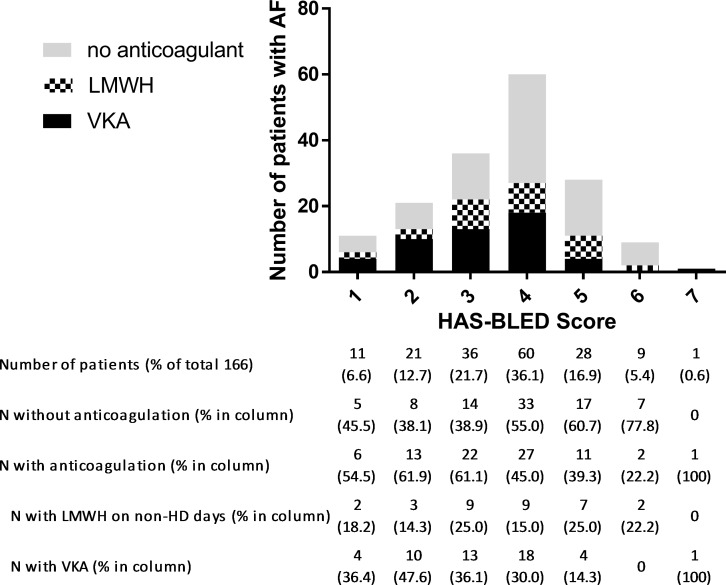
Distribution of patients with AF across HAS-BLED Scores and frequency of corresponding anticoagulation treatment.

In the AF group, 84 patients (50.6%) received anticoagulation treatment. 50 patients (30.1%) were orally anticoagulated with VKA, 33 patients (19.9%) received LMWH on non-HD days for long-term stroke prevention. One patient (0.6%) received fondaparinux in the same indication. Fifty-six patients (33.7%) received antiplatelet agents instead of anticoagulants for stroke prevention. Twenty-six AF patients (15.6%) received no antithrombotic therapy at all, except the anticoagulation during HD sessions. There was no association of CHA2DS2-VASc scores with use or non-use of anticoagulation therapy (p = 0.938) (Fig 3). Patients with higher HAS-BLED scores were less likely to receive anticoagulation therapy (p = 0.019) (Fig 4).

## Discussion

Patients with ESRD are at approximately 6-fold increased risk of stroke and systemic embolism compared to the general population [19–21]. One of the main culprits suspected of increasing the risk of stroke in ESRD patients is AF. In the cross-sectional evaluation of our population-based cohort study of 626 HD patients in Vienna, we found a 26.5% prevalence of AF.

Previous studies have determined the prevalence of AF in HD patients between 3.8 and 27% [9–13]. Studies with retrospective design or based on large insurance claims databases estimated a prevalence between 10 and 15% [13,14,22], while studies with prospective, patient-level data tended to find a higher prevalence of AF [11,12]. Prevalence rates derived from patient-level cohort studies can be suspected of selection bias, especially if a large number of centers contributes only few patients each, potentially over-representing AF patients. Nevertheless, from our findings we have to assume that the prevalence of AF is underestimated in large population-level cohorts. The data gathered in our investigation is entirely constituted of patient-level data, derived from personal patient interviews, structured review of the detailed medical records at the dialysis centers, and objective verification of AF based on standardized criteria. According to estimates from the Austrian Registry of Dialysis and Transplantation yearly report of 2013 [23], the cohort in our study covers ~ 73% of the population of HD patients in the city of Vienna (population 1.7 million).

In the general population and HD patients alike, AF is a disease of the elderly and is more frequent in men than in women [1,10,14]. In our cohort, the prevalence AF in patients 75 years of age and older reached over 40% and male sex was independently associated with 1.7-fold increased odds of AF diagnosis.

In search of other characteristics that may be associated with AF development in HD patients, we investigated the time on HD expressed as in HD age years. The risk of an AF diagnosis increased by 8% for every year a patient spent on HD treatment. We were further able to show that congestive heart failure was associated with AF diagnosis. Further cardiovascular conditions, which are generally considered risk factors for the development of AF, such as coronary heart disease, hypertension or valvular diseases, were not associated with AF. This may prove difficult in an HD cohort enriched with higher frequencies of comorbidities than the general population. It may be a specific characteristic of HD cohorts that recognized factors associated with AF in the general population may not apply. Surprisingly however, the risk of an AF diagnosis was independently increased in patients with a history of VTE. We ruled out a confounding relationship of catheter-associated or shunt-related thrombosis by only including non-catheter DVT and PE in the analysis. The interpretation of this novel relationship between a history of VTE and AF in HD patients will certainly require prospective analysis. The prevalence of AF was further associated with presence or history of malignancy. A causal connection through cardiotoxic cancer-therapy would have to be examined in a randomized trial setting. Prospective observational data could reveal if shared risk factors lead to confounding of the relationship between cancer and AF.

The high risk of stroke and bleeding in ESRD patients on HD and a complex risk profile including many risk factors for thromboembolic complications warrants a specific risk evaluation for AF patients on HD. The CHADS_2_, CHA_2_DS_2_-VASc, and HAS-BLED Scores, predict the risk of stroke and bleeding in the general population based on epidemiologic distribution of risk factors [24–26]. These risk evaluation approaches also have validity in HD patients [27–29], but because of the higher frequencies of risk factors amongst HD patients, these scores may lose the ability to distinguish between truly high and low risk patients. On the CHA_2_DS_2_-VASc scale 95.8% of the patients with AF in this cohort reached indication for continuous anticoagulation. Strict adherence to these generally established risk scores may lead to more aggressive anticoagulation treatment in HD patients. More recently, evidence from a Taiwanese nationwide cohort indicated that the risk of stroke in HD patients with AF may be overestimated [3] and that ethnicity may be a risk modifying factor. Cohorts of HD patients with AF are further never untreated cohorts, as the majority of patients receive LMWH during HD sessions to prevent clotting in the dialysis tubes.

There is currently no consensus on the appropriate use of antithrombotic agents for stroke prevention in HD patients. This is because patients on HD were excluded from trials on stroke prevention and not all results from non-randomized clinical studies show the same benefit of anticoagulation treatment for AF patients [30–34]. The treatment practices in our cohort showed 30.1% of AF patients receiving oral anticoagulant with VKA and 19.9% receiving LMWH on non-HD days for stroke prevention. Treatment practices from different cohorts had 25 to 58% use of VKA in AF patients, indicating that the prescription is very dependent on local preferences [8,28,34–38]. Due to renal elimination, LMWHs are essentially contraindicated in patients with a creatinine clearance < 30ml/min [39]. However, in the absence of evidence for a clear benefit of VKA in stroke prevention for AF, LMWH in prophylactic dose given on non-HD days may be an option in some cases of patients with very high risk of stroke. Despite lack of evidence for LMWH treatment as long-term stroke prevention in AF, almost 20% of AF patients in our cohort received this treatment. Over 50% of both AF and non-AF patients in this cohort received antiplatelet agents and treating physicians chose a cautious approach with LMWH over VKA in patients with indication for dual pathway antithrombotic therapy and tended to decide against anticoagulation in patients with higher HAS-BLED scores.

The present study has limitations in need of acknowledgment. The recruited cohort has a relatively small sample size, compared to national or insurance-claims registry studies and may thus be biased for the true prevalence of AF and associated medical conditions. However, this study was designed to capture an entire regional population of HD patients. We performed a cross-sectional analysis of 626 HD patients from the seven major HD centers in the city of Vienna and avoided a selection bias by recruiting a representative sample of approximately 73% of the regional HD population. The study design further ensured that the AF diagnosis derived from patient-level data, according to reproducible criteria for diagnosis. Asymptomatic, paroxysmal AF may, however, have been underdiagnosed. Since the HD centers are all within the city limits of Vienna, our cohort may represent a largely urban population and may therefore differ from more rural areas. Previous studies on Austrian populations of HD patients had similar demographic characteristics but lower prevalence rates of AF and lower use of VKA anticoagulants [22,36]. The risk of stroke and mortality associated with AF may lead to a decreased prevalence of AF in patients with long time on HD, otherwise known as healthy user bias. We cannot exclude that this form of selection bias may have led to further underestimation of the AF prevalence in our current analysis.

In conclusion, we confirmed that the prevalence of AF is high amongst ESRD patients on chronic hemodialysis. Prevalence data from retrospective and registry cohorts may underestimate the burden of AF in ESRD patients. The use of antithrombotic agents for stroke prevention in AF in our cohort indicates a lack of consensus for ideal antithrombotic therapy and therapy-guiding risk evaluation for thromboembolic complications and bleeding in HD patients may require more HD-specific risk factors.
